# Systematic Review and Meta-Regression of Factors Affecting Midline Incisional Hernia Rates: Analysis of 14 618 Patients

**DOI:** 10.1371/journal.pone.0138745

**Published:** 2015-09-21

**Authors:** David C. Bosanquet, James Ansell, Tarig Abdelrahman, Julie Cornish, Rhiannon Harries, Amy Stimpson, Llion Davies, James C. D. Glasbey, Kathryn A. Frewer, Natasha C. Frewer, Daphne Russell, Ian Russell, Jared Torkington

**Affiliations:** 1 University Hospital of Wales, Cardiff, CF14 4XW, United Kingdom; 2 Royal Gwent Hospital, Newport, NP20 2UB, United Kingdom; 3 Morriston Hospital, Heol Maes Eglwys, Swansea, SA6 6NL, United Kingdom; 4 Glan Clwyd Hospital, Rhyl, LL18 5UJ, United Kingdom; 5 Cardiff University School of Medicine, Cardiff, CF14 4XN, United Kingdom; 6 Swansea University College of Medicine, Swansea, SA2 8AA, United Kingdom; Heinrich-Heine-University and University Hospital Duesseldorf, GERMANY

## Abstract

**Background:**

The incidence of incisional hernias (IHs) following midline abdominal incisions is difficult to estimate. Furthermore recent analyses have reported inconsistent findings on the superiority of absorbable versus non-absorbable sutures.

**Objective:**

To estimate the mean IH rate following midline laparotomy from the published literature, to identify variables that predict IH rates and to analyse whether the type of suture (absorbable versus non-absorbable) affects IH rates.

**Methods:**

We undertook a systematic review according to PRISMA guidelines. We sought randomised trials and observational studies including patients undergoing midline incisions with standard suture closure. Papers describing two or more arms suitable for inclusion had data abstracted independently for each arm.

**Results:**

Fifty-six papers, describing 83 separate groups comprising 14 618 patients, met the inclusion criteria. The prevalence of IHs after midline incision was 12.8% (range: 0 to 35.6%) at a weighted mean of 23.7 months. The estimated risk of undergoing IH repair after midline laparotomy was 5.2%. Two meta-regression analyses (A and B) each identified seven characteristics associated with increased IH rate: one patient variable (higher age), two surgical variables (surgery for AAA and either surgery for obesity surgery (model A) or using an upper midline incision (model B)), two inclusion criteria (including patients with previous laparotomies and those with previous IHs), and two circumstantial variables (later year of publication and specifying an exact significance level). There was no significant difference in IH rate between absorbable and non-absorbable sutures either alone or in conjunction with either regression analysis.

**Conclusions:**

The IH rate estimated by pooling the published literature is 12.8% after about two years. Seven factors account for the large variation in IH rates across groups. However there is no evidence that suture type has an intrinsic effect on IH rates.

## Introduction

Incisional hernias (IHs) are defined as “abdominal wall gaps around postoperative scars, perceptible or palpable by clinical examination or imaging” [1, 2]. They are a common complication of midline closure following abdominal surgery, cause significant morbidity, impair quality of life, and are costly to treat [3, 4]. Patient risk factors associated with a higher incidence (usually described as a higher “rate”) of IHs include diabetes mellitus [5], obesity [5, 6], cachexia [7], increasing age [6], male sex [6, 8], chronic obstructive pulmonary disease (COPD) [7, 9], history of (or operation for) an abdominal aortic aneurysm (AAA) [10], anaemia [7], smoking [8], and corticosteroids [11]. Surgical characteristics associated with greater IH formation include urgent surgery [12, 13], layered rather than mass closure [12, 14], and interrupted rather than continuous suture closure [15], whilst use of closure adjuncts such as prophylactic mesh may reduce IH rates [16]. Despite assessment by several meta-analyses, the effect of suture type (absorbable versus non-absorbable) on IH rates is not clear [13, 17–19]; and unsurprisingly suture preference varies from surgeon to surgeon. Identification of IHs may also depend on length of follow up [12, 20–22], and the use of radiological investigations in combination with clinical examination for diagnosis, rather than clinical examination alone [23–25].

The reported incidence of IHs after midline laparotomy ranges from 0 to 44%, reflecting the heterogeneity of patients, surgery and follow up. This variation makes service planning for IH repair difficult, and also hinders the design of randomised controlled trials (RCTs). The aims of this review were therefore threefold: firstly, to estimate a pooled IH rate following surgery via a midline laparotomy as derived from the published literature; secondly, to identify factors which can account for the wide variability in IH reporting; and thirdly, to examine the effect of suture type (absorbable versus non-absorbable) on preventing the occurrence of IHs.

## Methods

We undertook a systematic review in accordance with PRISMA guidelines (see S1 File).[26] A detailed protocol and data abstraction proforma is available at https://wworth.swan.ac.uk/1624.aspx.

### Search strategy

We (D.C.B., J.A., I.T.R. and J.T.) designed a search strategy with the help of a specialist librarian (see S2 File for MeSH terms used). We (J.C.D.G., K.A.F. and N.C.F.) searched Medline and Embase via Ovid, PubMed, the Cochrane Central Register of Controlled Trials and the Cochrane Database of Systematic Reviews from January 1980 until March 2013. There was no restriction on publication type. We checked the references of included publications for other relevant papers.

### Paper selection

Two reviewers (from T.A., J.A., J.C., L.D., R.H., A.S. and D.C.B.) independently screened each title and abstract. Another two of these reviewers retrieved and independently screened potentially relevant full papers; an experienced surgeon resolved discrepancies (J.T.). We included full papers published in English if they described a population of adult patients undergoing primary suture closure of a midline laparotomy wound, and reported number of IHs and average length of follow-up (mean or median). We excluded papers describing IH repair, non-midline abdominal incisions, or closure by methods other than primary sutures (e.g. prophylactic mesh placement or metal sutures), and papers which did not report length of follow-up were excluded. We included papers reporting patients with both midline and non-midline wounds only if they reported data on midline incisions separately. Randomised trials, quasi-experiments, cohort studies and case series were all eligible for inclusion. We compared multiple publications from single datasets, and used the most complete used for abstraction.

### Data abstraction

We designed a proforma for data abstraction, piloted it on five papers, and refined it with input from all ten reviewers (D.C.B, T.A., J.A., J.C., R.H., A.S., L.D., J.C.D.G., K.A.F. and N.C.F.). Two reviewers (from T.A., J.A., J.C., R.H., A.S. and D.C.B.) independently abstracted data from each included paper; an experienced surgeon resolved discrepancies (J.T.). If papers reported IH rate and duration of follow-up separately for different patient groups (e.g. in RCTs with separate treatment arms), each patient group had data abstracted separately. We abstracted study characteristics (including exclusion criteria), patient demographics and co-morbidities, type of surgical procedure undertaken, closure method, suture type, duration of follow up and number of IHs (S1 Table). We considered IHs present if assessed clinically or radiologically in accordance with consensus guidelines [2]. When papers reported attrition of patients due to mortality or loss to follow up, we used the number of patients at follow-up, rather than enrolment, as the denominator.

### Quality assessment

We used the check list devised by Downs and Black to assess methodological quality [27]. This checklist can score both RCTs and observational studies on five methodological criteria: reporting (ten questions, eleven points), external validity (three questions, three points), bias (seven questions, seven points), confounding (six questions, eight points) and power (two questions, five points), with a maximum score of 34 (S3 File).

### Statistical analysis (D.R., I.T.R and D.C.B.)

We collected and analysed all data in SPSS^®^ version 20 (SPSS, Chicago, Illinois, USA). We summarised continuous data by means or medians, using the mean if both were available. We weighted these by number of patients to estimate IH rates. We derived confidence intervals (CI) from weighted T-tests or regression output. We used the Excel macro at: http://www.apho.org.uk/resource/view.aspx?RID=47241 to create funnel plots.

For meta-regression analysis we imputed missing variables by substituting weighted means [28]. We subtracted these weighted means from individual data for each variable to analyse data more accurately. We converted categorical study-level variables into binary variables (S1 Table). We weighted regression analyses by number of patients using the ‘weighted least squares’ function in SPSS^®^.

We regressed all study characteristics separately against IH rate to select variables for inclusion in meta-regression models. To avoid omitting characteristics significant only in combination with other variables, we used a significance level of 20% to select candidates for the multivariable models. We undertook two complementary meta-regression analyses ('stepwise' and 'backwards elimination').

## Results

### Overview

**Fig 1:**
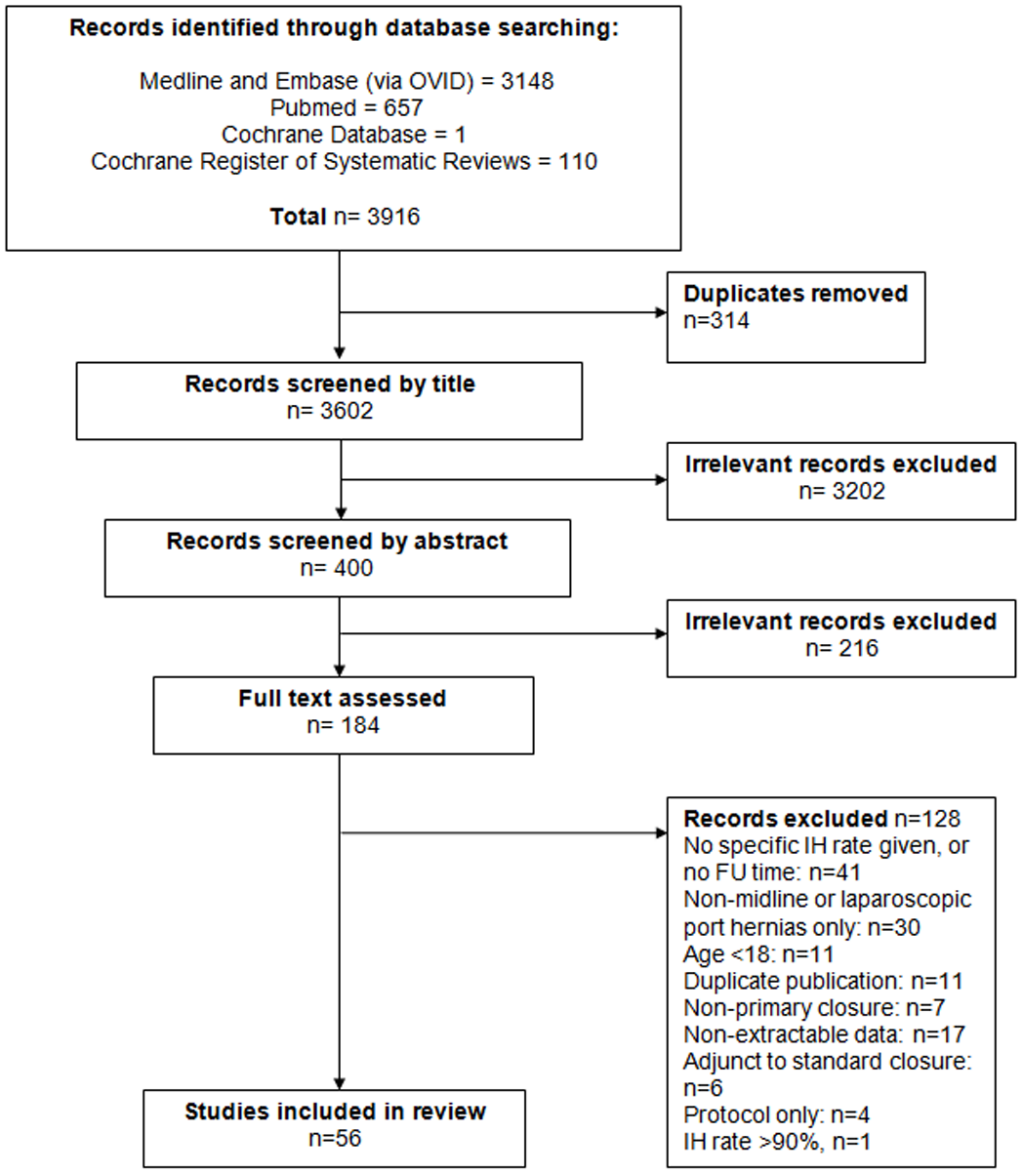
PRISMA diagram detailing search strategy and study selection process.

The initial search yielded 3602 unique publications, of which 184 papers were retrieved for full review. We judged 56 (27 RCTs, 21 cohort studies, four quasi-experiments and four case series) eligible for inclusion (Fig 1). Several papers yielded abstractable data for more than one treatment arm generating 83 separate patient groups comprising 14,618 patients for analysis (Table 1). Fourteen RCTs and 15 cohort studies provided data for only a single patient group, for example by comparing midline with transverse laparotomy. Downs and Black scores ranged from 8 to 31 with a median of 21. Excluded papers included 11 duplicate publications [29–39] and one paper which met the inclusion criteria but reported an IH rate of 91% (20 of 22 patients) [40], which we excluded as an extreme outlier.

**Table 1:** Characteristics of included studies including Downs and Black quality scores [27].

Study	Year	Type of study	Data analysis	Diagnosis of IH	Number of surgeons or institutions	Consecutive patients?	Group Number	Number of pts	Number of IHs (%)	Follow-up (months): mean (default) or median	Downs & Black score [27]
Guillou [63]	1980	RCT	Prospective	Clinical	Single institution	Yes	1	58	4 (6.9)	12	20
Bucknall [50]	1981	RCT	Prospective	Clinical	Single institution	Yes	1	83	9 (10.8)	8.4	22
Cormon [52]	1981	RCT	Prospective	NR	Single institution	Yes	1	49	4 (8.2)	19	20
Bucknall [64]	1982	Cohort study	Prospective	Clinical	Single institution	Yes	1	544	48 (8.8)	24	15
Shepherd [65]	1983	Cohort study	Prospective	NR	Single institution	Yes	1	200	10 (5.0)	24	11
Cox [66]	1986	RCT	Prospective	Clinical	Multiple institutions	Yes	1	159	20 (12.6)	12	20
McNeill [56]	1986	RCT	Prospective	NR	NR	NR	1	51	5 (9.8)	18	21
Playforth [67]	1986	Case series	Prospective	Clinical	Single surgeon	No	1	56	6 (10.7)	30^a^	8
Cameron [53]	1987	RCT	Prospective	Clinical	Single institution	Yes	1	100	10 (10.0)	14.7	25
							2	90	11 (12.2)		
Krukowski [55]	1987	RCT	Prospective	Clinical	Single institution	Yes	1	285	22 (7.7)	12	23
							2	295	28 (9.5)		
Paes [68]	1987	RCT	Prospective	NR	Single institution	Yes	1	51	2 (3.9)	15.2	17
Wissing [42]	1987	RCT	Prospective	Clinical	Multiple institutions	Yes	1	286	48 (16.8)	12	24
							2	290	60 (20.7)		
							3	281	37 (13.2)		
							4	299	31 (10.4)		
Schoetz [69]	1988	Cohort study	Prospective	Clinical	Single institution	Yes	1	172	5 (2.9)	12	14
Khaikin [70]	1991	Cohort study	Retrospective	Clinical	Single institution	Yes	1	31	1 (3.2)	10^a^	18
Trimbos [71]	1992	RCT	Prospective	Clinical	Multiple institutions	NR	1	122	7 (5.7)	12	24
							2	118	5 (4.2)		
Israelsson [57]	1994	Quasi-expt.	Prospective	Clinical	Single institution	Yes	1	325	49 (15.1)	12^a^	21
							2	318	50 (15.7)		
Carlson [54]	1995	RCT	Prospective	Clinical	Multiple institutions	Yes	1	91	4 (4.4)	24	18
							2	80	7 (8.8)		
Gislason [72]	1995	RCT	Prospective	Clinical	NR	Yes	1	412	30 (7.3)	12	22
Sivam [61]	1995	Quasi-expt.	Prospective	NR	Single institution	Yes	1	358	14 (3.9)	12.3	13
Brolin [51]	1996	RCT	Prospective	Clinical	Single surgeon	NR	1	109	20 (18.3)	28.3	14
							2	120	11 (9.2)	30.4	
Sugerman [41]	1996	Case series	Retrospective	Clinical	Single institution	NR	1	842	168 (20.0)	12	17
							2	162	7 (4.3)		
Colombo [73]	1997	RCT	Prospective	Clinical	Single institution	Yes	1	308	32 (10.4)	21	29
							2	306	45 (14.7)		
							3	53	2 (3.8)		
							4	59	0 (0.0)		
Adye [10]	1998	Cohort study	Retrospective	Clinical	Single institution	No	1	58	18 (31.0)	12	18
							2	42	5 (11.9)		
Mingoli [12]	1999	Case series	Retrospective	Clinical	Single institution	Yes	1	138	25 (18.1)	11.2	17
Hsiao [74]	2000	RCT	Prospective	Clinical	Single surgeon	Yes	1	93	5 (5.4)	24^a^	22
							2	71	0 (0.0)		
Musella [75]	2001	Cohort study	Retrospective	Clinical and radiological	NR	NR	1	51	16 (31.4)	48.6	19
							2	63	11 (17.5)		
Lai [76]	2002	Case series	Retrospective	NR	Single institution	Yes	1	19	3 (15.8)	27.3	9
Strzelczyk [77]	2002	Quasi-expt.	Prospective	Clinical	NR	Yes	1	48	9 (18.8)	12	13
Winslow [78]	2002	RCT	Prospective	Clinical	NR	NR	1	46	9 (19.6)	30.1	21
Lim [79]	2003	Cohort study	Prospective	NR	Single institution	Yes	1	92	2 (2.2)	20	23
Raffetto [80]	2003	Cohort study	Prospective	Clinical	Multiple institutions	Yes	1	177	50 (28.2)	30.8	21
							2	82	9 (11.0)	36.8	
Liapis [81]	2004	Cohort study	Prospective	NR	NR	Yes	1	197	32 (16.2)	63.7	16
							2	67	5 (7.5)	63.7	
Marwah [82]	2005	RCT	Prospective	NR	Single institution	Yes	1	50	15 (30.0)	6	13
Ihedioha [83]	2008	Cohort study	Prospective	Clinical	Single institution	Yes	1	63	10 (15.9)	22^a^	17
Laurent [84]	2008	Cohort study	Prospective	NR	Single institution	Yes	1	165	46 (27.9)	51^a^	22
Singh [85]	2008	Cohort study	Prospective	Clinical	Single institution	Yes	1	74	13 (17.6)	21.9	19
Togo [86]	2008	Cohort study	Retrospective	Clinical or radiological	Single institution	No	1	95	6 (6.3)	52.8	23
El-Khadrawy [87]	2009	RCT	Prospective	Radiological	Single institution	NR	1	20	3 (15.0)	36.3	20
Halm [88]	2009	RCT	Prospective	Clinical	Single institution	Yes	1	63	9 (14.3)	12^a^	29
Milbourn [89]	2009	RCT	Prospective	Clinical	Single institution	Yes	1	272	49 (18.0)	12	30
							2	250	14 (5.6)	12	
Seiler [15]	2009a	RCT	Prospective	Clinical and radiological	Multiple institutions	NR	1	176	28 (15.9)	12	31
							2	178	15 (8.4)		
							3	176	22 (12.5)		
Seiler [90]	2009b	RCT	Prospective	Clinical and radiological	Single institution	NR	1	79	13 (16.5)	12	27
Veljkovic [91]	2009	Cohort study	Prospective	Clinical	Single institution	No	1	603	81 (13.4)	6.9	24
Al-Dahamasah [92]	2010	Cohort study	Prospective	Clinical and radiological	Single institution	NR	1	284	16 (5.6)	20.6	17
Berretta [93]	2010	RCT	Prospective	Clinical and radiological	Single institution	NR	1	63	6 (9.5)	36	25
							2	63	4 (6.3)		
							3	65	7 (10.8)		
Bevis [16]	2010	RCT	Prospective	Clinical and radiological	Multiple institutions	Yes	1	45	16 (35.6)	20.3	22
Skipworth [94]	2010	Cohort study	Prospective	Clinical	Single institution	Yes	1	167	10 (6.0)	36^a^	13
Bloemen [23]	2011	RCT	Prospective	Clinical or radiological	Single institution	Yes	1	223	45 (20.2)	34.5	30
							2	233	58 (24.9)	33.3	
deSouza [95]	2011	Cohort study	Retrospective	Clinical or radiological	Single institution	Yes	1	142	28 (19.7)	21.2	25
							2	231	37 (16.0)	18.5	
Justinger [96]	2011	Quasi-expt.	Prospective	Clinical or radiological	Single institution	Yes	1	399	56 (14.0)	36^a^	21
							2	389	59 (15.2)		
Klarenbeek [97]	2011	RCT	Prospective	NR	Multiple institutions	Yes	1	52	2 (3.8)	6	23
Llaguna [98]	2011	Cohort study	Prospective	Clinical	Single surgeon	NR	1	62	11 (17.7)	17.7	19
Salayta [99]	2011	Cohort study	Prospective	Clinical or radiological	Single institution	NR	1	284	16 (5.6)	24	23
Albertsneier [100]	2012	RCT	Prospective	Clinical and radiological	Multiple institutions	NR	1	112	21 (18.8)	12	25
Gruppo [43]	2012	Cohort study	Prospective	Clinical	Single institution	Yes	1	412	51 (12.4)	67.2	20
							2	653	73 (11.2)	75.6	
Lee [101]	2012	Cohort study	Prospective	Clinical or radiological	Single institution	No	1	68	20 (29.4)	28.2^a^	18

^a^: Median

Quasi-expt. Quasi-experimental study

NR Not reported

Note For RCTs or observational studies with more than one group, we specify individual patient numbers, IH rates and follow-up time for each group; for some RCTs or observational studies, however, we abstracted only one group, either because only one met the inclusion criteria, or because we could not abstract two groups independently.

### Incisional hernia rates

**Fig 2:**
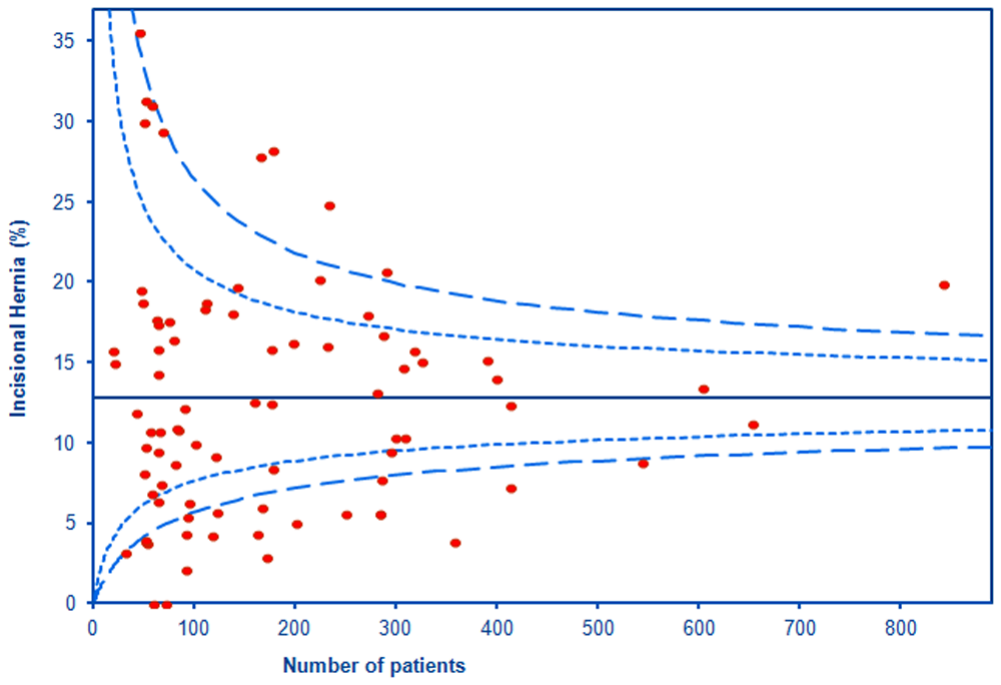
Funnel plot of IH rates (y axis) by number of patients in study (x axis). **Notes**: Created using Excel macro at www.apho.org.uk/resource/view.aspx?RID=47241. Dashed boundaries show ± three standard errors; feint show ± two standard errors.

The mean IH rate was 12.8% (SD 7.7%; 95% CI: 11.4 to 14.2%) at a weighted mean follow-up time of 23.7 months. The funnel plot in Fig 2 shows a symmetrical spread of data around the mean, but greater than would be expected if the underlying IH rate were constant. The largest patient group (with 842 patients) had an IH rate substantially above the expected range [41]; these patients all underwent gastric bypass surgery for morbid obesity, with thus a greater predicted IH rate. The two largest studies enrolled 1156 and 1065 patients, with IH rates of 15.3% [42] and 11.7% [43] respectively. Both would fall within the boundary in Fig 2 showing two standard errors, but do not appear at these points because data were abstracted as four and two groups respectively.

### Study characteristics and incisional hernia rates

IH rates were comparable between: RCTs and non-RCTs (12.3 versus 13.2%; 95% CI for difference: -3.8 to 1.8%; p = 0.49); papers reporting consecutive patients or not (12.6 versus 14.8%; 95% CI for difference: -7.9 to 3.6%; p = 0.46); and studies enrolling elective patients or elective and emergency patients (13.1 versus 13.0%; 95% CI for difference: -3.1 to 3.3%; p = 0.95). Retrospective studies reported significantly greater IH rates than prospective studies (17.3 versus 12.1%; 95% CI for difference: 1.2 to 9.2%; p = 0.012). IH rates were greater, but not significantly greater, in studies that included patients with previous IHs (15.3 versus 12.7%; 95% CI for difference: -1.0 to 6.1%; p = 0.15) and patients on steroids (14.9 versus 11.6%; 95% CI for difference: -1.3 to 7.9%; p = 0.16). Studies that included patients with previous laparotomies had a significantly greater IH rate (15.0 versus 11.5%; 95% CI for difference: 0.1 to 6.9%; p = 0.043). IH rates detected clinically were similar to those diagnosed clinically or radiologically (12.614.6%; 95% CI for difference: -5.1 to 1.1%; p = 0.22).

**Fig 3:**
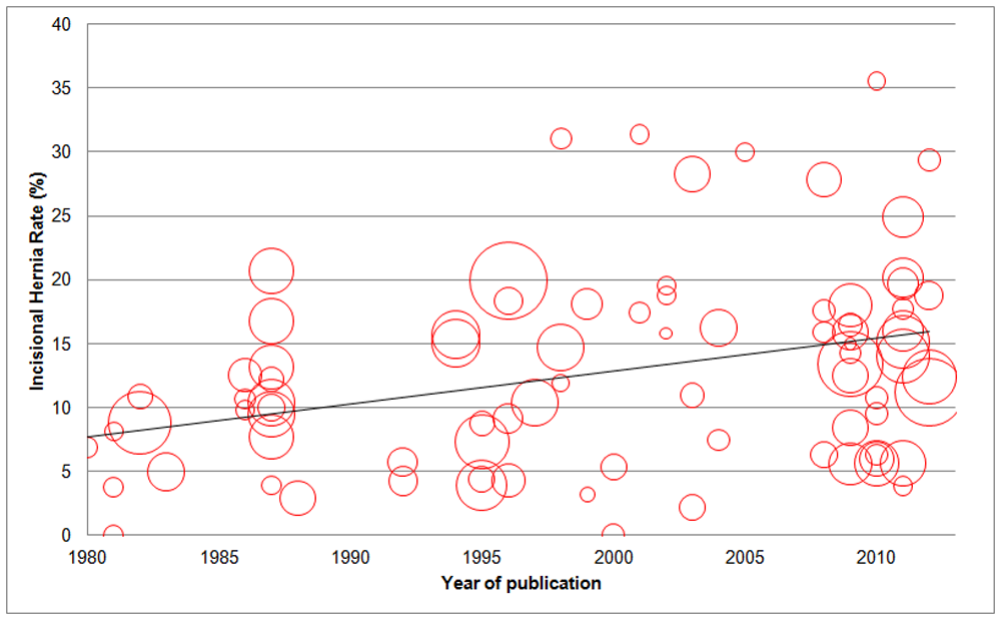
Bubble plot of IH rates by year of publication. **Notes**: The area of each circle is proportionate to the number of patients. The line of best fit shows that IH rates increase with year of publication.

We used year of publication as a proxy for date of surgery: reported IH rates increased with year of publication (Table 2 and Fig 3; p = 0.033). Duration of follow up was significantly longer in non-RCTs than in RCTs (29.2 months versus 16.8 months; p = 0.001). Nevertheless this had no significant effect on reported IH rates (p = 0.59). Downs and Black scores also did not predict IH rates.

**Table 2:** Univariable analysis of IH rates.

**Continuous (patient level) variables**	**Number of included groups (patients)**	**Weighted mean**	**Number of zero value papers: groups (patients)**	**Coefficient B (SE)**	**95% CI for B**	**Univariable significance level**
Males	38 (5761)	39.5%	11 (1876)	10.71 (3.41)	3.94 to 17.49	0.002
Gynaecological surgery	51 (7672)	23.6%	41 (5859)	-6.57 (2.16)	-10.88 to -2.25	0.003
AAA surgery	47 (6968)	10.6%	42 (6255)	8.69 (3.17)	2.38 to 14.99	0.008
Age (mean or median)	57 (9370)	58.7 years	0 (0)	0.20 (0.76)	0.049 to 0.35	0.010
Lower midline incision	40 (6026)	27.4%	28 (4006)	-5.65 (2.58)	-10.79 to -0.51	0.031
Year of publication (from 1980)	83 (14146)	19.9 years	1 (58)	0.16 (0.67)	0.12 to 0.28	0.033
Upper midline incision	40 (6026)	26.1%	25 (4210)	5.45 (2.53)	0.42 to 10.47	0.034
Vascular surgery	49 (7216)	25.6%	37 (5318)	3.90 (2.26)	-0.59 to 8.38	0.088
**Categorical (study level) variables**	**Number of included groups (patients)**			**Coefficient B (SE)**	**95% CI for B**	**Univariable significance level**
	**Total**	**Yes (score 1)**	**No (score 0)**			
Prospective (vs. retrospective) data collection	83 (14146)	71 (12744)	12 (1874)	-5.21 (2.02)	-9.24 to -1.18	0.012
Obesity surgery	83 (14146)	7 (1283)	76 (13335)	5.28 (2.42)	0.47 to 10.09	0.032
Includes patients with previous laparotomies	46 (9913)	17 (3912)	29 (6001)	3.51 (1.70)	0.12 to 6.90	0.043
Includes patients with existing IHs	41 (8931)	19 (4328)	22 (4603)	2.59 (1.78)	-0.95 to 6.12	0.150
Includes patients on steroids	40 (6439)	9 (1278)	31 (5161)	3.29 (2.31)	-1.30 to 7.88	0.158
**Downs & Black [27] criteria (study level)**	**Number of included groups (patients)**			**Coefficient B (SE)**	**95% CI for B**	**Univariable significance level**
	**Total**	**Yes (score 1)**	**No (score 0)**			
Similar follow up between groups	83 (14146)	70 (12157)	13 (2461)	6.01 (1.76)	2.51 to 9.51	0.001
Appropriate statistical analyses	83 (14146)	65 (11878)	18 (2740)	5.74 (1.69)	2.38 to 9.09	0.001
Exact significance levels specified	83 (14146)	70 (12744)	13 (1874)	6.24 (1.99)	2.28 to 10.19	0.002
Outcomes clearly described	83 (14146)	75 (13045)	8 (1573)	6.29 (2.16)	1.99 to 10.59	0.005
Sufficient follow up	83 (14146)	76 (13593)	7 (1025)	6.63 (2.66)	1.35 to 11.91	0.015
Outcomes measured with a valid test	83 (14146)	72 (13006)	11 (1612)	5.32 (2.17)	1.01 to 9.63	0.016
Sufficient data given	83 (14146)	52 (9887)	31 (4731)	3.02 (1.47)	1.00 to 5.93	0.043
Clear hypothesis	83 (14146)	77 (13454)	6 (1164)	4.85 (2.54)	-0.21 to 9.91	0.060
Recruits representative of sample population	83 (14146)	73 (12550)	10 (2068)	-3.40 (1.98)	-7.34 to 0.55	0.091

### Regression analyses

S2 Table lists the study characteristics which we abstracted and specifies the binary variables into which we disaggregated categorical variables. Twenty-two of these achieved the significance level of 20% to become candidates for the meta-regression models. Table 2 shows the results of regressing IH rate on each of these. Significance levels before and after imputing missing data were very similar, with an identical choice of variables for the multivariable meta-regression analyses.

We undertook two complementary pre-specified meta-regression analyses using backward elimination and stepwise regression (Table 3, models A and B). Each model identified seven significant study or patient characteristics that together predicted higher IH rates (including six common variables and one of two others): five apparently causal—inclusion of patients with previous laparotomies, inclusion of patients with previous IHs, higher mean (or median) age of patients, surgery for AAA and either surgery for obesity (model A) or upper midline incision (model B); and two circumstantial—later year of publication, and reporting exact significance levels. Both models significantly improved on models with fewer predictors, and achieved impressive, and very similar, adjusted R^2^ (0.403 and 0.404 respectively).

**Table 3:** Regression analyses of IH rates on multiple predictors.

Variables (in order of significance level)	Coefficient B (SE)	95% CI for B	Significance level
**Model A—backwards elimination**			
Includes patients with previous laparotomies	6.09 (1.49)	3.12 to 9.05	<0.001
Exact significance levels specified	4.93 (1.73)	1.49 to 8.38	0.006
Age (mean or median)	0.20 (0.072)	0.057 to 0.35	0.007
Year of publication (from 1980)	0.16 (0.064)	0.029 to 0.28	0.017
Obesity surgery	4.86 (2.03)	8.90 to 0.82	0.019
AAA surgery	6.43 (2.80)	0.84 to 12.01	0.025
Study includes patients with existing IHs	3.01 (1.49)	0.042 to 5.98	0.047
**Model B—stepwise**			
Includes patients with previous laparotomies	6.02 (1.49)	3.05 to 9.00	<0.001
Exact significance levels specified	5.17 (1.72)	1.74 to 8.59	0.004
Age (mean or median)	0.20 (0.072)	0.053 to 0.34	0.008
Year of publication (from 1980)	0.16 (0.064)	0.028 to 0.28	0.017
Upper midline incision	5.23 (2.16)	0.93 to 9.53	0.018
AAA surgery	6.62 (2.81)	1.01 to 12.20	0.021
Includes patients with existing IHs	2.66 (1.52)	-0.37 to 5.68	0.084

Notes: Model A: significance level for exclusion = 5%

Model B: significance level for inclusion = 10%; significance level for exclusion = 12%

### The effect of suture material on IH rates

Almost all papers provided data on type of suture used for midline closure, yielding a subset of 75 patient groups comprising 13,157 patients, of whom 25.5% received non-absorbable sutures, 56.2% slowly absorbable, and 18.3% rapidly absorbable. Univariable analysis showed no significant difference (p = 0.54) between absorbable and non-absorbable sutures (IH rates of 13.5 and 11.9% respectively; 95% CI for difference: -2.0 to 5.1%). Forcing suture type into either multivariable model did not affect other regression coefficients, and suture type remained non-significant. Several sensitivity analyses failed to find any subpopulation where suture type affects IH rates (Table 4). Rapidly absorbed sutures showed the highest IH rate (15.6%), but not significantly higher than either slowly absorbable (13.0%; 95% CI for difference: -1.6% to 6.9%; p = 0.234), or non-absorbable (11.9%; 95% CI for difference: -1.7 to 8.9%; p = 0.170) sutures, consistent with published analyses [13, 17].

**Table 4:** Univariable regression of suture type (absorbable or non-absorbable) on IH rates.

Analysis	Number of groups (patients)	Significance level	B (95% CI for B)
All studies	75 (13157)	0.544	-1.06 (-4.54 to 2.41)
Randomised trials	45 (6485)	0.925	0.20 (-4.11 to 4.51)
Multiple site studies	17 (2724)	0.881	0.61 (-7.92 to 9.14)
Includes previous laparotomy	29 (6001)	0.929	-0.28 (-6.60 to 6.04)
Includes previous IH	22 (4603)	0.818	-0.77 (-7.70 to 6.15)
Includes emergency surgery	35 (7383)	0.927	0.23 (-4.78 to 5.24)
Continuous closure	59 (9875)	0.901	0.28 (-4.14 to 4.70)
Studies with comparative data^a^	51 (10441)	0.900	0.02 (-3.83 to 4.35)
Downs & Black score ≥21	46 (8888)	0.619	1.12 (-3.38 to 5.61)

^a^ Studies with more than one patient group available for analysis.

Summary Suture type (absorbable versus non-absorbable) had no effect on IH rates.

### Other outcomes

Of those with IHs, 49.0% (95% CI: 18.4 to 79.6%) were symptomatic, and 36.0% (95% CI: 21.1 to 50.9%) underwent IH repair. The risk of patients requiring IH repair after a midline laparotomy was 5.2% (95% CI: 2.8 to 7.7%). The use of non-absorbable sutures had no effect on the likelihood of IHs being symptomatic or undergoing repair (p = 0.95 and p = 0.49 respectively). Stitch sinuses occurred in 1.8% of patients (95% CI: 0.8 to 2.9%); these were more likely, but not significantly more likely, with non-absorbable suture material (p = 0.057). Wound infections occurred in 8.7% of patients, but these did not affect the incidence of IHs (p = 0.22).

## Discussion

This systematic review and meta-regression of 14,618 patients from 83 patient groups has demonstrated a weighted mean IH rate of 12.8% at a weighted mean of 23.7 months follow-up after surgery via a midline laparotomy. Approximately one half of IHs are symptomatic; and about one third undergo repair. The risk of needing further surgery for IH after a midline incision is approximately 5%.

Our search strategy sought all available evidence on the epidemiology of IH, notably by including all recognised research designs, both randomised and not. Although trials generally provide the best evidence for evaluating effectiveness, they are less well suited to assessing risk factors; they tend, not only to have narrow inclusion criteria, but also to restrict length of follow-up. Fortunately our rigorous analysis generated well-behaved statistical models characterising the influence of a range of methodological, patient and surgical variables. In particular, though the mean duration of follow-up in trials (16.8 months) was significantly shorter than in other designs (29.2 months), the mean IH rate was very similar (12.3% versus 13.2%).

Two consistent meta-regression models have each identified seven independent factors associated with increased IH rate—one patient variable (higher age), two surgical variables (surgery for AAA and either surgery for obesity surgery (model A) or using an upper midline incision (model B)), two inclusion criteria (including patients with previous laparotomies and those with previous IHs), and two circumstantial variables (later year of publication and specifying an exact significance level). Suture type had no effect on IH rates.

To our knowledge this meta-regression is the only such analysis of midline abdominal incisions to date. Data abstraction was preferentially at the time of outpatient assessment, rather than patient enrolment, thus excluding early post-operative mortality and loss to follow up, thereby giving a more clinically relevant rate. Several studies excluded established high-risk groups, including those on steroids or with previous IHs. This suggests that an unselected cohort more representative of day-to-day surgical practice would suffer an even greater incidence of IHs.

The patient variables we identified as associated with IHs are consistent with previous reports. Increasing age is known to be a risk factor for IHs [6], as is bariatric surgery [5, 6] and a history of (or operation for) an AAA [44]. The use of an upper-midline incision has not been studied in isolation as a risk factor for IHs; as it was significantly correlated with bariatric surgery, however, these incisions may act as a proxy for open obesity surgery. Several patient variables were significant in other studies but not here, for example male sex [6, 8] (significant only in univariable analysis), or a history of diabetes [5] (not significant at any stage). Although postoperative infection has previously shown correlation with increased IH rates [21], this study showed no such association. The absence of all these variables from the final model has three possible explanations: they are correlated with other significant predictors; they are not reported in all studies, or otherwise difficult to abstract; or meta-regression can distort relationships because it averages patient characteristics within single data points [45].

The later the year of publication, the more reported IH rates increased. There are many plausible explanations for this association: operating on patients at greater risk of IHs; more rigorous follow up and diagnosis; better reporting over time; or gradual change in surgical technique. Nevertheless IHs appear more prevalent in modern surgical practice than previously.

In both univariable and multivariable analyses, reporting exact significance levels (rather than reporting a result as “not significant” or “p less than” a specified value) was associated with significantly higher IH rates. Whilst surprising that a simple change from vague to specific probability statement had such an effect, especially as the Downs and Black quality score had no effect on IH rates, it was a highly significant variable in both regression models. It may be that this variable is simply a proxy for methodological and reporting rigour, similar to other such ‘effect modifiers’ noted in previous meta-regression analyses [46]. This finding highlights the value of standardised significance level reporting in the literature.

The length of follow up had no apparent effect on IH rates. This finding is contrary to previous publications showing that rates at one year underestimate the overall burden of IHs. For example Fink’s review of 775 patients enrolled in two RCTs showed IH rates increased from 12.6% at one year to 22.4% at three years [22]. Similarly Hoer *et al*. followed patients for ten years, and found 54% of IH developed after twelve months, 75% after two years and 89% after five years [6]. However our meta-regression did not show this effect, probably because we had to analyse data grouped by study, rather than individual data; so other differences between studies may have obscured the effect of duration of follow up. The estimated IH rate herein corresponds with an average follow-up time of approximately two years. According to Hoer *et al*. [6], about 75% of IH would be clinically apparent at this point. This equates with an IH rate of approximately 17% at ten years. While early studies may underestimate the long-term incidence of IH, IHs that develop later are generally smaller and cause few symptoms [47].

Despite numerous RCTs and several meta-analyses, there is little consensus in choosing between absorbable and non-absorbable sutures for midline closure [48]. Addressing this issue is useful, as suture type is more readily altered than many other variables. Both have potential problems: absorbable sutures lose their tensile strength with time and thus fail to support marginal scar tissue; whereas non-absorbable sutures have a theoretically greater risk of “buttonholing” the rectus sheath by repeated ‘sawing’ through the fascia with abdominal wall movement [49]. RCTs have reported conflicting results on reducing IHs: some favour non-absorbable sutures [42, 50]; others favour absorbable sutures [51, 52], but most show no difference [23, 53–57]. Meta-analyses also report conflicting results: Weiland *et al*. (eight trials; n = 3607 including non-midline incisions) [19], Rucinski *et al*. (fifteen trials; n = 5851) [58] and Hodgeson *et al*. (sixteen trials; n = 5028) [18], found non-absorbable sutures better at reducing IH rates. In contrast Salid *et al*. (eight trials; n = 4261) [59], Van Riet *et al*. (five trials of slowly absorbing versus non-absorbing material; n = 2669) [17] and Diener *et al*. (six trials of emergency and elective patients n = 3219) [13] found no difference in IH rates with suture type. Our meta-regression has confirmed that suture material does not affect IH rates whether analysed alone or in combination with other significant factors. However there was a non-significant tendency for non-absorbable sutures to increase the rate of suture sinuses. As neither material reduces IH formation, surgeons may prefer slowly absorbable sutures [60] to reduce post-operative pain [20] and suture sinus formation [17, 23].

Finally our analysis unequivocally identifies patient groups at high risk of IH:, elderly patients; those undergoing AAA or obesity surgery; and patients with previous laparotomies or IHs. Though our review did not have the power to identify the best treatment for these minority groups, we conclude that they need special consideration and possible change in technique, for example prophylactic placement of mesh or more complex forms of suture closure such as the 'Hughes repair’ (also known as the ‘Cardiff near-and-far” or ‘Smead-Jones’ repair) [61, 62].

## Conclusions

IHs are an increasingly reported problem in surgical practice, with an estimated rate of 12.8% in published studies. This rate is likely to be greater in general surgical practice. Factors affecting reported IH rates include patient characteristics, surgical characteristics, inclusion criteria, and circumstantial reporting factors. However there is no evidence that absorbable and non-absorbable sutures differ in their effects on IH rates.

## Supporting Information

S1 File PRISMA checklist. (DOCX)

S2 File Search criteria used. (DOCX)

S3 File Quality scoring system by Downs and Black [27]. (DOCX)

S1 Table Complete list of variables extracted from each paper, typically as percentages. (DOCX)
