# Supplementary material for: Systematic Review and Meta-Regression of Factors Affecting Midline Incisional Hernia Rates: Analysis of 14 618 Patients
Source: PLoS One. 2015 Sep 21;10(9):e0138745. doi: 10.1371/journal.pone.0138745 (PMC4577082; doi:10.1371/journal.pone.0138745)
Supplement: S2 File — (DOCX) [file pone.0138745.s002.docx]

**S2 File. Search criteria used**

PubMed:

((Incisional OR Ventral OR Abdominal [mh]) AND (midline OR laparotomy [mh])) AND (hernia [mh] OR herniation [mh])

Medline and Embase via Ovid:

((Incisional OR Ventral OR Abdominal [mh]) AND (midline OR laparotomy [mh])) AND (hernia [mh] OR herniation [mh])

The Cochrane Central Register of Controlled Trials and the Cochrane Database of Systematic Reviews:

(incisional OR ventral OR abdominal) AND (midline OR laparotomy) AND (hernia OR herniation)
