# Supplementary material for: Systematic Review and Meta-Regression of Factors Affecting Midline Incisional Hernia Rates: Analysis of 14 618 Patients
Source: PLoS One. 2015 Sep 21;10(9):e0138745. doi: 10.1371/journal.pone.0138745 (PMC4577082; doi:10.1371/journal.pone.0138745)
Supplement: S3 File — (DOCX) [file pone.0138745.s003.docx]

**S3 File. Quality scoring system by Downs and Black**

**Reporting**

1 Is the hypothesis, aim or objective of the study clearly described?

Yes – 1 point, No – 0 points.

2 Are the main outcomes to be measured clearly described?

Yes – 1 point, No – 0 points.

3 Are the characteristics of the patients included in the study clearly described?

Yes – 1 point, No – 0 points.

4 Are the interventions of interest (i.e. closure method of midline laparotomy) clearly described?

Yes – 1 point, No – 0 points.

5 Are the distributions of principal confounders (including prognostic factors that are considered to be potential confounders, i.e. risk factors for IH formation) in each group of subjects to be compared clearly described?

Yes – 2 points, Partially – 1 point, No – 0 points.

6 Are the main findings of the study clearly described?

Yes – 1 point, No – 0 points.

7 Does the study provide enough information to allow the reader to calculate estimates of the variability in the data for the main outcomes (i.e. standard error, standard deviation or confidence intervals (normally distributed data), confidence intervals, inter-quartile range, minimum and maximum (non-normally distributed data))?

Yes – 1 point, No – 0 points.

8 Have all important adverse effects that may be a consequence of the intervention been reported?

Yes – 1 point, No – 0 points.

9 Have the characteristics of patients lost to follow-up been described?

Yes – 1 point, No – 0 points.

10 Have confidence intervals or exact significance levels been reported for the main outcomes?

Yes – 1 point, No – 0 points.

**External validity**

11 Were the subjects asked to participate in the study representative of the entire population from which they were recruited (i.e. an unselected sample of consecutive patients)?

Yes – 1 point, No – 0 points.

12 Were those subjects who participated representative of the entire population from which they were recruited (requires information of the proportion of those who were asked to take part agreed to take part)?

Yes – 1 point, No – 0 points.

13 Were the staff, places, and facilities where the patients were treated, representative of the treatment the majority of patients receive?

Yes – 1 point, No – 0 points.

**Bias**

14 Was an attempt made to blind those measuring the main outcomes of the intervention?

Yes – 1 point, No – 0 points.

15 If any of the results of the study were based on “data dredging”, was this made clear?

Yes – 1 point, No – 0 points.

16 In trials and cohort studies, do the analyses adjust for different lengths of follow-up of patients (or are comparable within 10%)?

Yes – 1 point, No – 0 points.

17 Was the length of follow-up adequate (defined as a FU of 1 year or more)?

Yes – 1 point, No – 0 points.

18 Were the statistical tests used to assess the main outcomes appropriate?

Yes – 1 point, No – 0 points.

19 Was non-compliance reported appropriately?

Yes – 1 point, No – 0 points.

20 Were the main outcome measures used valid and reliable?

Yes – 1 point, No – 0 points.

**Confounding**

21 Were the patients in different intervention groups (trials and cohort studies), or the cases and controls (case-control studies), recruited from the same population?

Yes – 1 point, No – 0 points.

22 Were study subjects in different intervention groups (trials and cohort studies), or the cases and controls (case-control studies), recruited over the same period of time?

Yes – 1 point, No – 0 points.

23 Were study subjects randomised between interventions?

Yes, adequately – 2, Yes but method not described or not robust – 1, No or not reported – 0.

24 Was the randomised assignment concealed from both patients and health care staff until recruitment was complete and irrevocable?

Yes – 1 point, No – 0 points.

25 Did the analyses from which the main findings were drawn adjust for selection bias?

Yes – 2, Partially – 1, No or not reported – 0.

26 Were patients lost to follow-up noted?

Yes – 1 point, No – 0 points.

**Power**

27 Was a power calculation performed?

Yes, data-based – 2, Yes, based on clinical opinion etc – 1, No – 0.

28 Is the sample size adequate?

>200 – 3, >100 – 2, <100 but justified – 1, <100 and not justified – 0.
