# Supplementary material for: Systematic Review and Meta-Regression of Factors Affecting Midline Incisional Hernia Rates: Analysis of 14 618 Patients
Source: PLoS One. 2015 Sep 21;10(9):e0138745. doi: 10.1371/journal.pone.0138745 (PMC4577082; doi:10.1371/journal.pone.0138745)
Supplement: S1 Table — (DOCX) [file pone.0138745.s004.docx]

**S3 Table. Complete list of variables extracted from each paper, typically as percentages**

| **Study design** | **Exclusion criteria** | **Patient factors (Number of patients unless stated)** | **Surgical factors**  **(Number of patients unless stated)** | **Operative factors** |
| --- | --- | --- | --- | --- |
| Type of study  (RCT^a^, Quasi-experiment, Cohort or Case series) | Previous laparotomy  (Yes or No) | Sex | Urgency (emergency versus elective) | Type of procedure  (open or laparoscopic) |
| Number of patients | Previous IH  (Yes or No) | Age  (median, mean) | Cancer operation | Incision^b^ (upper midline, lower midline or upper & lower midline) |
| Consecutive patients  (Yes or No) | Immunosuppressed patients (Yes or No) | History of Diabetes Mellitus (DM) | Bariatric surgery | Contamination^b^ (clean, part contaminated, contaminated, dirty) |
| Data analysis (Retrospective or Prospective) | Current pregnancy (Yes or No) | History of congestive cardiac failure (CCF) | Colorectal surgery | Type of closure  (continuous or interrupted) |
| Definition of IH (Clinical^a^, Radiological, Clinical OR Radiological or Clinical AND Radiological) | Emergency surgery (Yes or No) | History of renal disease | Other GI surgery | Type of suture (rapidly absorbable, slowly absorbable  or non-absorbable^a^) |
| Number of surgeons  or institutions  (Single surgeon, Single institution, Multiple institutions**^a^**) |  | History of abdominal aortic aneurysm (AAA) | Vascular surgery | Closure suture length: wound length ratio^b^ <4, =4 or >4 |
| Follow up time |  | History of connective tissue disorder | Renal surgery | Closure bite size [large (>10mm) or small (<10mm)] |
|  |  | History of hepatic disease/jaundice | Gynaecological surgery |  |
|  |  | History of cancer | Other surgery |  |
|  |  | History of chronic obstructive pulmonary disease (COPD) |  |  |

Notes a The category compared with all others as a binary variable in regression analysis.

b All possible binary variables for inclusion in regression analysis were assessed, but none were significant.
